# Non-ischemic Painful Intermittent Left Bundle Branch Block With Infra-Hisian Block Treated Successfully With Biventricular Pacemaker: A Case Report and Literature Review

**DOI:** 10.7759/cureus.20907

**Published:** 2022-01-03

**Authors:** Rana Al-Zakhari, Safa Aljammali, Nicholas Sheets, Granit Veseli, Nidal Isber

**Affiliations:** 1 Internal Medicine, Richmond University Medical Center, New York, USA; 2 Electrophysiology, Northwell Health, New York, USA; 3 Electrophysiology, Richmond University Medical Center, New York, USA

**Keywords:** biventricular pacemaker, infra-hisian block, electrophysiology, angiographic coronary artery disease, ventricular desynchrony, intermittent left bundle branch block

## Abstract

Non-ischemic painful left bundle branch block (LBBB) is defined as chest pain that occurs simultaneously with the appearance of left bundle branch block and resolves with the disappearance of the left bundle branch block in patients without evidence of myocardial ischemia. The underlying mechanism of this rare clinical occurrence has not been fully understood, but it has been proposed that it results from ventricular dyssynchrony. In this case report, we present a 65-year-old male with non-ischemic chest pain who was found to have intermittent left bundle branch block (ILBBB) with infra-Hisian conduction delay, treated successfully with a biventricular pacemaker. After excluding the presence of angiographic coronary artery disease, an electrophysiology study was conducted to direct the management and investigate other causes of chest pain. The present study highlights the importance of obtaining electrophysiology studies in patients with painful left bundle branch block with no angiographic evidence of coronary artery disease to diagnose this uncommon syndrome.

## Introduction

Non-ischemic painful intermittent left bundle branch block (ILBBB) is an uncommon conduction disturbance that is often a marker for cardiac disease. The prevalence of ILBBB during exercise stress tests (EST) has been found to be 0.38% by Stein et al., but other investigators suggest it could be as high as 1.1% [1,2]. Patients present with varying symptoms, from mild and self-limiting chest discomfort to severe, debilitating pain with exertion or even at rest. This clinical entity is thought to be underdiagnosed, especially in the setting of coexisting coronary artery disease [1]. Herein, we report a case of chest pain associated with ILBBB in a patient with no angiographic evidence of obstructive coronary artery disease. The patient was found to have an infra-hisian block and was treated successfully with a biventricular pacemaker.

## Case presentation

A 65-year-old male with a history of hypertension presented with a complaint of angina on activity. He received a coronary stent for 70% stenosis of his left anterior descending artery. The patient had no relief from symptoms despite optimal medical therapy, which included beta blockers and antianginal medications. One year later, his symptoms progressed, with angina occurring daily and triggered by minimal activity, such as "walking to the kitchen." He also started to have symptoms after meals. He described the chest pain as retrosternal, non-radiating, and associated with shortness of breath and palpitations. His angina would last for a few minutes and force the patient to stop and rest. As his condition progressed, the pain became debilitating, which prompted a coronary angiogram that showed patent coronary arteries with no obstructive lesions.

**Figure 1:**
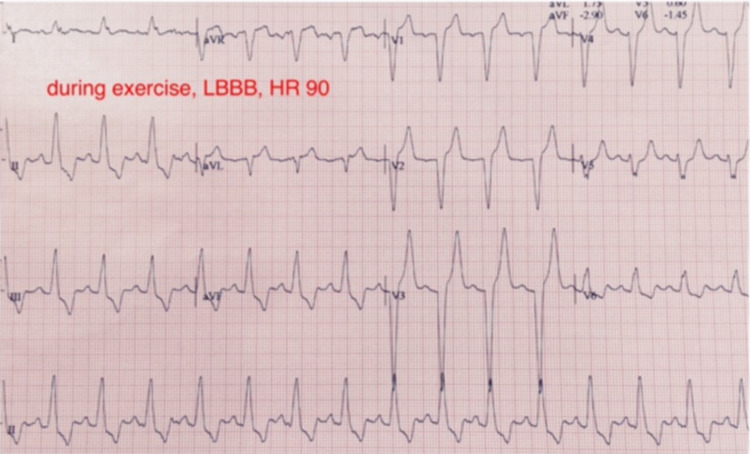
Intermittent left bundle branch block induced by exercise.

**Figure 2:**
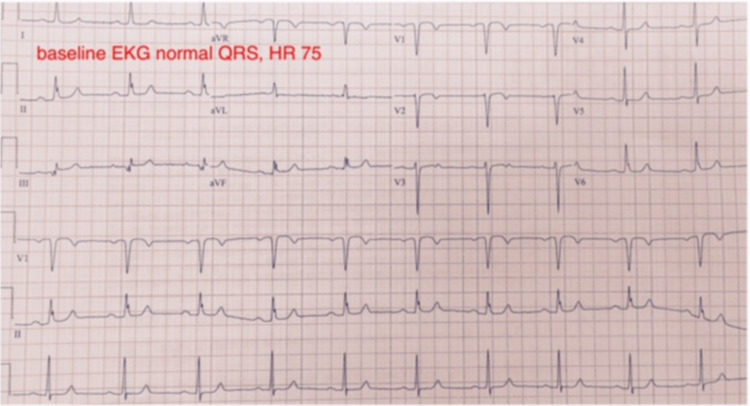
EKG at baseline and post exercise.

The EST revealed the development of chest pain with the simultaneous appearance of LBBB at a heart rate of 90 beats per minute, which occurred only one minute after the onset of exercise (Figure 1). With cessation of exercise, the chest pain resolved simultaneously with the resolution of LBBB (Figure 2).

**Figure 3:**
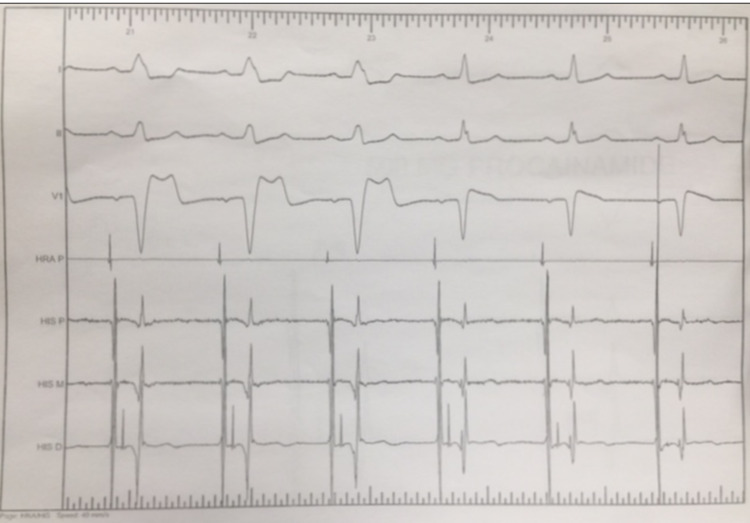
Spontaneous intermittent left bundle branch block during electrophysiology study.

**Figure 4:**
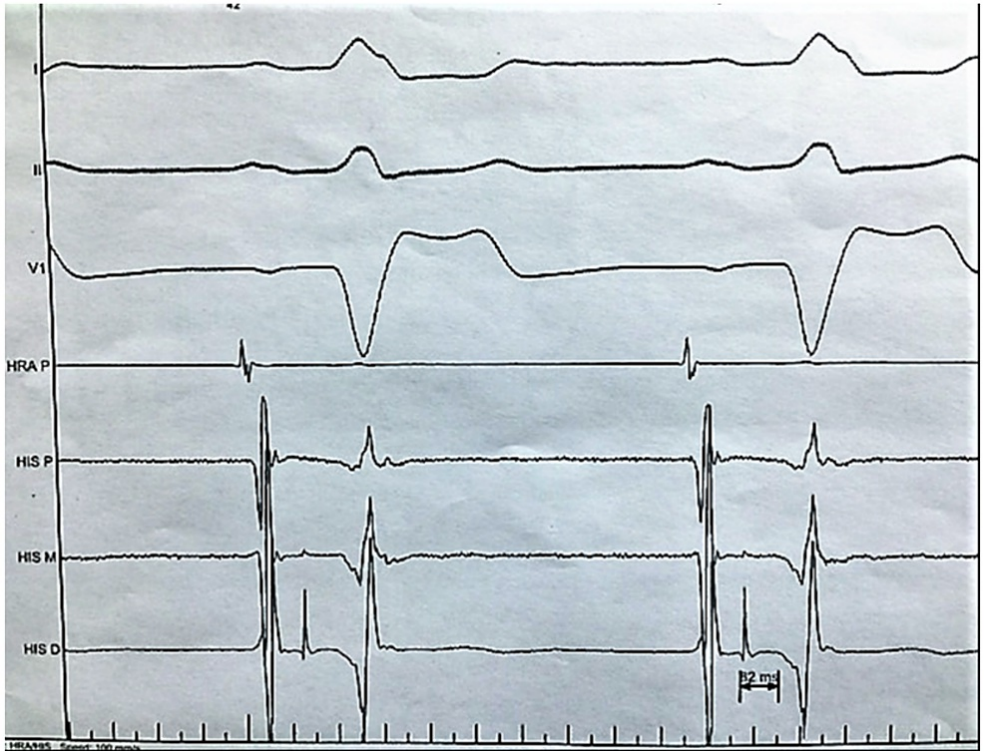
Baseline H-V interval prolonged to 82 ms reflecting infra-Hisian conduction disease.

Myocardial perfusion tests showed no inducible ischemia. An echocardiogram showed no segmental wall motion abnormalities, a normal ejection fraction of 60%, and no valvular lesions. A 12 lead EKG at rest showed normal sinus rhythm with no abnormalities. An electrophysiology study was performed to assess the AV conduction reserve. Intermittent LBBB develops spontaneously and during atrial pacing at any pacing atrial rate faster than the sinus rate (Figure 3). The HV interval at baseline was moderately prolonged at 80 msec (Figure 4).

**Figure 5:**
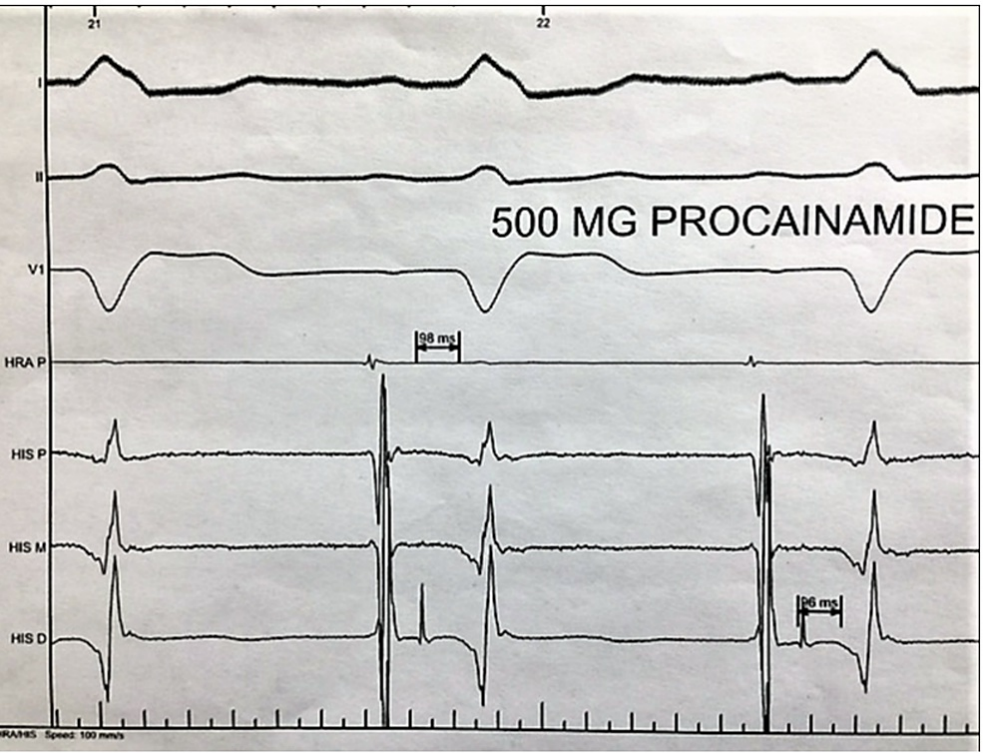
After Procainamide infusion H-V interval is progressively longer to 96 ms suggesting poor infra-Hisian conduction reserve.

After infusion of 500 mg of procainamide, the HV became severely prolonged at 96 ms with no infra-hisian conduction block (Figure 5). The tilt table test was negative for orthostatic hypotension. The patient’s symptoms were confirmed to be related to the development of LBBB on EST and, because of the EP study finding of infra-Hisian conduction disease, the patient received a biventricular pacemaker that resulted in complete resolution of his symptoms.

## Discussion

LBBB results from a degeneration of the conduction system or is a reflection of myocardial pathology. Complete LBBB is characterized by a QRS duration of 120 ms or more, predominantly upright complexes with broad-slurred R waves in leads I and V6, a QS or rS complex in lead V1, and a monophasic R wave in leads I, V5, and V6 [2]. The main causes of new onset LBBB include coronary artery disease, cardiomyopathies, and hypertension [3]. ILBBB results from a transient or an episodic blockade at a specific point of the conduction system. The alteration of the heart rate is considered the most common cause of ILBBB, which is usually caused by phase 3 block or tachycardia-dependent block when a stimulus reaches the tissues that are still in the refractory period [3,4]. Exercise-induced ILBBB is a rare phenomenon that occurs in 0.1% to 1.1% of patients undergoing diagnostic EST [2]. It has been reported that the onset of ILBBB at a heart rate greater than 125 bpm is highly consistent with normal coronary arteries, but it has been noticed to occur at a slower rate of <105 bpm, like the presentation of our patient [5].

Painful ILBBB is an uncommon variant that results in chest pain that appears simultaneously with the development of LBBB and occurs with or without myocardial ischemia. The prevalence of this condition in the general population is still unknown, as it is uncommon and frequently underrecognized owing to coexisting coronary artery disease [6]. The underlying mechanism of rate-dependent ILBBB and chest pain in patients with normal coronary arteries is not well understood. However, the dyssynchronous ventricular contraction during the appearance of ILBBB has been the predominant theory for the origin of the chest pain. In addition, the presence of microcirculatory ischemia undetectable by coronary angiography and the interoceptive sensitivity of the central nervous system might be contributing factors [7]. However, the immediate onset/offset character of the chest pain is inconsistent with ischemia. Bory et al. demonstrated that nitroglycerin has failed to relieve pain and, in some cases, resulted in the development of painful LBBB by inducing reflex tachycardia. However, they concluded that not all instances of painful LBBB are related exclusively to tachycardia and that myocardial ischemia is not a likely underlying mechanism [8]. Negative nuclear imaging and coronary sinus lactate sampling have been shown to be inconsistent with ischemia [8,9]. In addition, resolution of the chest pain on conversion from intermittent to permanent LBBB argues against ischemia [9]. On the basis of the observation of the cases that have been reported in the literature, Shvilkin et al. have suggested specific criteria to aid in the diagnosis of this phenomenon (Table 1) [9]. Shvilkin et al. also suggested criteria regarding the reversible T-wave inversion reported with ILBBB favoring cardiac memory against myocardial ischemia (Table 2) [9].

**Table 1:** Criteria for the diagnosis of painful non-ischemic intermittent left bundle branch block.

1. Abrupt onset of the chest pain concomitant with the appearance of the LBBB.
2. Simultaneous resolution of symptoms with resolution of LBBB (although cases have reported with a walk-through phenomenon; the chest pain resolved before the disappearance of LBBB).
3. Normal 12- lead ECG before and after LBBB (occasionally T-wave inversions maybe present and consistent with cardiac memory).
4. No evidence of myocardial ischemia. Normal left ventricular function.
5. Low S/T wave ratio (<2.5) in the precordial leads mainly V2-V3 favors the recent onset of LBBB with inferior QRS axis, sensitivity (100%) and specificity (89%).

**Table 2:** T-wave inversion criteria favoring cardiac memory against myocardial ischemia (92% sensitivity, 100% specificity).

1. Lead AVL: positive T-wave.
2. Lead I: positive/isoelectric T-wave.
3. Precordial leads: maximum T-wave inversion > T-wave inversion in Lead III.

Our case involves a patient with chest pain who did not respond to revascularization with a stent placement and with optimal medical therapy. His chest pain was caused by the development of rate-dependent LBBB. Ischemia is not likely the cause of LBBB, but instead, it is the presence of intrinsic infra-Hisian conduction disease. LBBB develops at a critical rate secondary to increased refractory period in the conduction system. LBBB and chest pain occurred at rest or after eating when the heart rate only minimally increased, excluding ischemia, especially in the absence of obstructive coronary arteries and the absence of ischemia on a nuclear stress test.

Regarding management, no specific guidelines are available currently as only case reports have been published in the literature. It was reported by Heinsimer et al. that increasing the heart rate of LBBB onset/offset from 110 to 175 bpm with a submaximal aerobic exercise regimen for three months resulted in a resolution of pain during daily activities. The benefit was limited by the recurrence of symptoms once the patient stopped the exercise [10]. Attenuating the heart rate response to exercise by beta blockers, thus preventing the heart rate from reaching the critical level that is associated with the development of LBBB, has been reported, but its benefit has not been consistently demonstrated and might result in worsening of symptoms by decreasing the heart rate at LBBB onset [11]. Our patient was already on a beta-blocker. It seems that the development of symptoms with rate-related LBBB is due to intrinsic infra-Hisian conduction disease and did not respond to beta blockers. After ruling out the presence of coronary artery disease, an electrophysiology study must be performed using atrial pacing or isoproterenol to correlate the chest pain with the onset of LBBB to localize the level of blockade and to determine the benefit of implanting a permanent pacemaker or attempting CRT using Biv-pacing or direct LBB pacing if the blockade is found to be distal to achieve ventricular resynchronization. In the literature, four cases with painful ILBBB have been treated with a dual chamber pacemaker and His bundle pacing [12-14]. Our patient was diagnosed with ILBBB with infra-Hisian block, which necessitated the use of a biventricular pacemaker to control his symptoms and significantly improve his quality of life.

## Conclusions

The present case and review of the literature highlights the importance of diagnosing this rare clinical phenomenon, despite its uncommon occurrence in the general population. After excluding the presence of angiographic coronary artery disease, an EP study must be conducted to direct the management toward conduction system pacing that will restore ventricular contraction and completely control the symptoms. With this case report, we hope to demonstrate that in the absence of angiographic coronary artery disease, there should be a high index of suspicion for ILBBB. We hope that this approach can guide the diagnosis and management of this rare presentation in future cases.
